# The Role of Human and Animal Monocytes and Macrophages in Homeostasis and Disease

**DOI:** 10.3390/ijms242216397

**Published:** 2023-11-16

**Authors:** Malgorzata Kloc, Jacek Z. Kubiak

**Affiliations:** 1 The Houston Methodist Research Institute, Transplant Immunology, Houston, TX 77030, USA; 2 The Houston Methodist Hospital, Department of Surgery, Houston, TX 77030, USA; 3 MD Anderson Cancer Center, Department of Genetics, The University of Texas, Houston, TX 77030, USA; 4 Laboratory of Molecular Oncology and Innovative Therapies, Military Institute of Medicine—National Research Institute (WIM-PIB), Szaserow 128, 04-141 Warsaw, Poland; 5 Dynamics and Mechanics of Epithelia Group, Institute of Genetics and Development of Rennes, Faculty of Medicine, University of Rennes, CNRS, UMR 6290, 35043 Rennes, France

Monocytes and macrophages are the innate immune cells that are the first-line responders to invading pathogens or foreign objects. They are evolutionarily ancient cells with the amoeba-like ability to engulf and destroy their target. Although invertebrates do not have the typical monocytes or macrophages that vertebrates do, they have functionally and morphologically equivalent immune cells such as amebocytes, hemocytes, and coelomocytes [1,2]. 

In vertebrates, which have adaptive immunity in addition to their innate immune system, monocytes/macrophages signal to adaptive immune cells in order to activate or silence their immune responses. For decades it was believed that monocytes/macrophages, and their equivalents in invertebrates, simply carry out target destruction, but studies have indicated that they can develop short- and long-term memory, which work much like adaptative immunity, allowing them to remember a previous encounter with a pathogen and mount a rapid immune response during subsequent encounters with the same, or similar, pathogens [3,4]. In this issue, Ni et al. describe how macrophages interact with their surrounding cells and tissues in inflammation-related diseases, and how the inflammatory environment changes macrophage phenotypes. They also propose novel therapeutic methods which monitor and prevent the transition of macrophage phenotypes. Another article in this issue, by Lin and Hu, focuses on the role of macrophage infiltration in chronic kidney disease. They discuss how the tissue plasminogen activator (tPA), a serine protease regulating the homeostasis of blood coagulation and fibrosis signaling pathways, affects macrophage function in response to kidney injury. Other aspects of innate immune cell biology that have been discovered in recent years, undermining our simplistic understanding of their functions, are the phenotypical diversity of macrophages [5,6], tissue-specific macrophages [7], the role of tumor-associated macrophages (TAMs) in cancer development and metastasis [8], and the reciprocal interactions between macrophages and stem cells [9]. 

Recent studies indicate that invertebrates’ immune cells produce a wide range of antimicrobial peptides (AMPs) with an astonishingly broad spectrum of activity and high selectivity against various pathogens. Ladewig et al. [10] constructed cDNA expression libraries from a marine cnidarian and ctenophore—the moon jellyfish *Aurelia aurita* and the comb jelly *Mnemiopsis leidyi*—and screened them for AMPs, which can prevent opportunistic pathogens from forming biofilms. They identified five AMPs that were inhibitory against *Klebsiella oxytoca*, *Staphylococcus epidermidis*, *S. aureus*, and *Pseudomonas aeruginosa.* These results indicate that marine invertebrates could be an attractive source of novel antimicrobial agents. Using Drosophila’s innate immune system as a model for antiviral responses, Tramell et al. [11] studied the insulin-mediated signaling regulation of the mosquito-borne West Nile virus. They identified peptide endothelin signaling as a novel pathway for West Nile infection and demonstrated its applicability to the human immune system. These findings are highly significant as mosquito-borne arboviruses represent a significant threat to humans worldwide; they are also surprising because endothelins are vasoconstrictors, and thus regulate blood flow [11]. However, peptide endothelin signaling may have many other functions, such as the recently discovered regulation of sex differences in salt balance management and the kidney response to high salt intake [12]. In this Special Issue, Makjaroen et al. describe how lipopolysaccharide (LPS) tolerance might increase susceptibility to secondary infection and/or viral reactivation, and that, in cases of LPS tolerance, macrophages have reduced glycolysis and mitochondrial activities, decreased anti-viral responses, and a down-regulation of anti-viral genes, such as Mx1 and Isg15. Their mouse experiments demonstrated the virus reactivation and deficit in the anti-viral responses found in LPS tolerance, which are probably caused by the reduced energy status of macrophages.

One of the most important sources of microorganisms in animals and humans is the gut. In healthy organisms, the gut microbiome is separated from the host by the gut barrier. However, damage to the gut barrier allows pathogens or their molecules to infiltrate into the host. In this issue, Charoensappakit et al.’s review describes how the gut translocation of microbial molecules can induce systemic inflammation that is especially dangerous in patients with active lupus. They also discuss how an imbalance in the gut microbiota may initiate gut permeability and exacerbate lupus. 

The inflammatory properties of macrophages are the basis of not only lupus but many other diseases as well. Thus, many studies focus on finding novel anti-inflammatory compounds. In this issue, Moço et al. describe the synthesis of fourteen derivatives of a natural monoterpene, carvone, with anti-inflammatory properties. The testing of these compounds revealed that four of them reduced the level of LPS-induced nitric oxide synthase and reduced nitric oxide production. The authors used the Ligand Express drug discovery platform to predict the targets of these compounds and their intestinal and blood–brain barrier permeability. Although the tested compounds had relatively low anti-inflammatory potency and specificity, the authors believe that they could be a foundation for further chemical modification that may yield novel, highly effective anti-inflammatory drugs.

Another disease related to chronic inflammation is rheumatoid arthritis (RA). The synovial tissues of RA patients contain high levels of proinflammatory cytokines such as IL-26. The studies by Wang et al., in this issue, showed that murine and human macrophage stimulation with IL-26 induce the macrophage inflammatory cytokines IL-9 and IL-17A by increasing the expression of IRF4 and RelB. They also showed that IL-26 activates the AKT-FoxO1 pathway, and that the inhibition of AKT phosphorylation enhances the IL-26 stimulatory effects on macrophages. The authors concluded that IL-26 promotes IL-9- and IL-17-expressing macrophages and might initiate IL-9- and IL-17-related adaptive immunity processes in RA. They suggested targeting IL-26 as a novel therapeutic strategy for rheumatoid arthritis and other IL-9- and IL-17-dependent diseases.

In the study by Ribeiro et al. on macrophage-associated low-grade inflammation, macrophages were incubated with indoxyl sulfate at both low concentrations and concentrations found in uremic patients, either alone or with the LPS challenge. Indoxyl sulfate is a uremic toxin that accumulates in the blood of patients with chronic kidney disease (CKD) and is a predictor of morbidity and mortality. The authors showed that the exposure of macrophages to indoxyl sulfate induced reactive oxygen species and low-grade inflammation. Indoxyl sulfate combined with the LPS challenge increased TNF-α, CCL2, and IL-10 release without significantly affecting macrophage polarization. Pre-treatment with indoxyl sulfate, followed by the LPS challenge, induced the expression of the aryl hydrocarbon receptor (Ahr) and NADPH oxidase 4 (Nox4), and generated reactive oxygen species (ROS). The authors concluded that indoxyl sulfate induces low-grade inflammation, affects macrophage function, and increases the LPS-induced inflammatory response.

Another study in this Special Issue concentrated on the inflammatory role of macrophages in the development of glomerulosclerosis during diabetic nephropathy (DN). Torres-Arévalo et al. showed that MRS1754, a selective antagonist of the A2B adenosine receptor (A2BAR), which decreases glomerulosclerosis and macrophage–myofibroblast transition in rats and has anti-fibrotic effects, attenuated the upregulation of immune-related pathways in the glomeruli of DN rats, and decreased the glomerular expression/secretion of CCL2, CCL3, CCL6, and CCL21, the infiltration of macrophages, and their polarization to M2, suggesting the involvement of macrophages in the anti-fibrotic effect of MRS1754. 

Interestingly, macrophages are also involved in the development of various chronic diseases in response to psychological stress. Momčilović et al., in this issue, studied the contributions of macrophages and extracellular ATP, as an indicator of disturbed tissue homeostasis, to erythropoiesis under conditions of repeated psychological stress in a mouse model. They found that repeated stress increased the number of erythroid progenitor cells and CD71+/Ter119+ and CD71−/Ter119+ cells in the bone marrow and spleen of mice, and that it induced the expression of the P2X7 receptor (P2X7R) and ectonucleotidase CD39. Macrophage depletion ameliorated the effects of repeated stress, indicating their important role in the response to psychological stress.

One of the most fascinating subjects in macrophage/innate immune cell biology is the ability of these cells to generate immune memory [13]. Historically, such ability was believed to be limited to adaptative immune cells, such as T cells and B cells. Below, we present the evidence of innate immune memory in invertebrate animals. The innate immune memory was identified in 2003, when Kurtz and Franz described that the exposure of the crustacean *Macrocyclops albidus* to its natural tapeworm parasite induced a resistance to sequential infection by the same, or similar, parasites [14]. A similar resistance to reinfection was also described in the insects *Anopheles gambiae*, *Galleria mellonella*, *Tenebrio molitor*, Periplaneta americana, and the worm *C. elegans* [15,16,17,18,19]. A fascinating observation is that, in crustaceans and insects, the innate immune memory can be maternally transferred to subsequent generations. *Daphnia magna* transfers its immunity to *Pasteuria romosa* bacteria; the brine shrimp *Artemia franciscana* transfers resistance to Vibrio infection to the next generation; the *Tribolium castaneum* beetle transfers its resistance to *Bacillus thuringiensis* to the next two generations; and the honeybee *Apis mellifera*, primed with *Paenibacillus* larvae, produces offspring with an increased number of innate immune cell hemocytes [20,21,22,23,24,25]. The innate immune memory and the transgenerational transfer of immunity have been also described in vertebrate species [26,27]. A review article by Kloc et al., discussing recent findings on macrophage memory, is included in this Special Issue.

Although the major role of macrophages and monocytes is to protect against pathogens, tissue- and organ-specific macrophages have many other, pathogen-unrelated functions. One such function, common for many tissue-resident macrophages, is their role in wound healing and aging. The cardiac tissue, in addition to the macrophages derived from monocytes and recruited to the heart, has a specific subset of reparative macrophages that respond to myocardial injury caused by disease or aging [28]. On a similar theme, Kloc et al.’s review article describes the role of macrophage-derived and non-macrophage-derived foam cells in atherosclerosis. The lungs have their own subtype of macrophages, the alveolar macrophages, which not only protect the lungs from infection and non-biological allergen particles, but are also very selective sentinels of healthy state and do not respond to antigens that do not cause lung injury [29]. Kupffer cells are liver-specific macrophages that participate in the metabolism of proteins and lipids and the removal of dead cells. They also protect the liver from infection and have anti-cancer activities [30]. Recent studies indicate that, at least in mice, Kupffer cells prevent the metastasis of pancreatic cancer to the liver [31]. Interestingly, various tumors have their own tumor-associated macrophages (TAMs) that can stimulate angiogenesis, enhancing tumor survival, proliferation, motility, extravasation, and metastasis. TAMs are also immunosuppressive and prevent T cells and natural killer cells from attacking tumor cells [8].

Another area of interest is the multilevel, reciprocal interaction between macrophages and stem cells. This is especially important for the emerging applications of mesenchymal stem cells for therapeutic use [32]. Studies show that mesenchymal stem cells (MSCs) can communicate with macrophages, either via direct cell–cell contact, the secretion of soluble factors, or organelle transfer, and modulate macrophage proliferation, phagocytosis, and functional activation. In turn, macrophages can change the differentiation, migration, apoptosis, and immunomodulatory properties of MSCs [33].

Even from the short description above, it is evident that the study of macrophages is incredibly important for many areas of biology and medicine. Because invertebrates do not have an adaptive immune system, studies of invertebrate macrophages, in isolation and without interference from adaptive immunity processes, can provide invaluable insight into the ancestral function of innate immune cells and their evolution. Studies of organ- and tissue-specific macrophages can help improve our understanding of their organ-specific attributes and help us design macrophage-targeted organ-specific therapies. Studies of TAMs and the crosstalk between macrophages and stem cells will elucidate the effects of macrophages on stemness and open novel therapeutic avenues for many as yet incurable diseases.

## References

[1] Buchmann K. (2014). Evolution of Innate Immunity: Clues from Invertebrates via Fish to Mammals. Front. Immunol..

[2] Loker E.S., Adema C.M., Zhang S.M., Kepler T.B. (2004). Invertebrate immune systems—Not homogeneous, not simple, not well understood. Immunol. Rev..

[3] Melillo D., Marino R., Italiani P., Boraschi D. (2018). Innate Immune Memory in Invertebrate Metazoans: A Critical Appraisal. Front. Immunol..

[4] Santosa E.K., Sun J.C. (2023). Cardinal features of immune memory in innate lymphocytes. Nat. Immunol..

[5] Decano J.L., Maiorino E., Matamalas J.T., Chelvanambi S., Tiemeijer B.M., Yanagihara Y., Mukai S., Jha P.K., Pestana D.V.S., D’souza E. (2023). Cellular Heterogeneity of Activated Primary Human Macrophages and Associated Drug–Gene Networks: From Biology to Precision Therapeutics. Circulation.

[6] Frank A.S.J., Larripa K., Ryu H., Röblitz S. (2023). Macrophage phenotype transitions in a stochastic gene-regulatory network model. J. Theor. Biol..

[7] Mass E., Nimmerjahn F., Kierdorf K., Schlitzer A. (2023). Tissue-specific macrophages: How they develop and choreograph tissue biology. Nat. Rev. Immunol..

[8] Noy R., Pollard J.W. (2014). Tumor-associated macrophages: From mechanisms to therapy. Immunity.

[9] Dehnavi S., Sadeghi M., Tavakol Afshari J., Mohammadi M. (2023). Interactions of mesenchymal stromal/stem cells and immune cells following MSC-based therapeutic approaches in rheumatoid arthritis. Cell. Immunol..

[10] Ladewig L., Gloy L., Langfeldt D., Pinnow N., Weiland-Bräuer N., Schmitz R.A. (2023). Antimicrobial Peptides Originating from Expression Libraries of *Aurelia aurita* and *Mnemiopsis leidyi* Prevent Biofilm Formation of Opportunistic Pathogens. Microorganisms.

[11] O’Brien M.W., Shivgulam M.E. (2023). Mechanistic, participant, and movement-related factors that contribute to low-flow-mediated constriction. Eur. J. Appl. Physiol..

[12] Nasci V.L., Almutlaq R.N., Pollock D.M., Gohar E.Y. (2023). Endothelin mediates sex-differences in acclimation to high salt diet in rats. Biol. Sex Differ..

[13] Sharrock J., Sun J.C. (2020). Innate immunological memory: From plants to animals. Curr. Opin. Immunol..

[14] Kurtz J., Franz K. (2003). Innate defence: Evidence for memory in invertebrate immunity. Nature.

[15] Rodrigues J., Brayner F.A., Alves L.C., Dixit R., Barillas-Mury C. (2010). Hemocyte Differentiation Mediates Innate Immune Memory in *Anopheles gambiae* Mosquitoes. Science.

[16] Bergin D., Murphy L., Keenan J., Clynes M., Kavanagh K. (2006). Pre-exposure to yeast protects larvae of Galleria mellonella from a subsequent lethal infection by Candida albicans and is mediated by the increased expression of antimicrobial peptides. Microbes Infect..

[17] Faulhaber L.M., Karp R.D. (1992). A diphasic immune response against bacteria in the American cockroach. Immunology.

[18] Moret Y., Siva-Jothy M.T. (2003). Adaptive innate immunity? Responsive-mode prophylaxis in the mealworm beetle, *Tenebrio molitor*. Proc. R. Soc. B Biol. Sci..

[19] Zhang Y., Lu H., Bargmann C.I. (2005). Pathogenic bacteria induce aversive olfactory learning in Caenorhabditis elegans. Nature.

[20] Tran T.D., Luallen R.J. (2024). An organismal understanding of *C. elegans* innate immune responses, from pathogen recognition to multigenerational resistance. Semin. Cell Dev. Biol..

[21] Little T.J., Killick S.C. (2007). Evidence for a cost of immunity when the crustacean *Daphnia magna* is exposed to the bacterial pathogen *Pasteuria ramosa*. J. Anim. Ecol..

[22] Low C.F., Chong C.M. (2020). Peculiarities of innate immune memory in crustaceans. Fish Shellfish. Immunol..

[23] Roy S., Baruah K., Bossier P., Vanrompay D., Norouzitallab P. (2022). Induction of transgenerational innate immune memory against Vibrio infections in a brine shrimp (*Artemia franciscana*) model. Aquaculture.

[24] Schulz N.K.E., Sell M.P., Ferro K., Kleinhölting N., Kurtz J. (2019). Transgenerational Developmental Effects of Immune Priming in the Red Flour Beetle Tribolium castaneum. Front. Physiol..

[25] Hernández López J., Schuehly W., Crailsheim K., Riessberger-Gallé U. (2014). Trans-generational immune priming in honeybees. Proc. Biol. Sci..

[26] Boraschi D., Italiani P. (2018). Innate Immune Memory: Time for Adopting a Correct Terminology. Front. Immunol..

[27] Roth O., Beemelmanns A., Barribeau S.M., Sadd B.M. (2018). Recent advances in vertebrate and invertebrate transgenerational immunity in the light of ecology and evolution. Heredity.

[28] Li J., Xin Y., Wang Z., Li J., Li W., Li H. (2023). The role of cardiac resident macrophage in cardiac aging. Aging Cell.

[29] Hussell T., Bell T.J. (2014). Alveolar macrophages: Plasticity in a tissue-specific context. Nat. Rev. Immunol..

[30] Nguyen-Lefebvre A.T., Horuzsko A. (2015). Kupffer Cell Metabolism and Function. J. Enzymol. Metab..

[31] Thomas S.K., Wattenberg M.M., Choi-Bose S., Uhlik M., Harrison B., Coho H., Cassella C.R., Stone M.L., Patel D., Markowitz K. (2023). Kupffer cells prevent pancreatic ductal adenocarcinoma metastasis to the liver in mice. Nat. Commun..

[32] Afkhami H., Mahmoudvand G., Fakouri A., Shadab A., Mahjoor M., Komeili Movahhed T. (2023). New insights in application of mesenchymal stem cells therapy in tumor microenvironment: Pros and cons. Front. Cell Dev. Biol..

[33] Lu D., Xu Y., Liu Q., Zhang Q. (2021). Mesenchymal Stem Cell-Macrophage Crosstalk and Maintenance of Inflammatory Microenvironment Homeostasis. Front. Cell Dev. Biol..

